# Rubber Dam

**Published:** 1874-06

**Authors:** 


### Editorial.

### RUBBER DAM.

Of all the instruments and appliances used in operations upon the natural teeth, none accomplish the object for which they are intended better than *Rubber Dam*. It has become a necessity in the performance of operations upon the natural teeth, espec ally in an approach to the present standard of perfection. It renders feasible and easy some operations that could not be done at all without it, and gives great facility of execution in all the more common operations. And he who would keep abreast with the foremost in the profession must use Rubber Dam.

As in everything else there is a great difference in ability to use it with care and efficiency by different persons. Some have not availed themselves of proper instruction in its use, some imagine that the bare mention, or a brief description at most, is sufficient for them, and in their efforts go on blundering indefinitely. How much better it would be, to go at once and obtain the information requisite, to make it most available. A journey of a thousand miles with the money and time requisite, would be well devoted for procuring such information, as would enable one to use rubber dam efficiently.

In the May number of *Johnston's Dental Miscellany* the first article will be found, as good a description of the method of using *Rubber Dam*, as has anywhere appeared, indeed we do not remember to have seen it so well described before; yet there are some dentists, who from so perfect a description would fail to get the application; some people make their eyes more available in obtaining knowledge than any other organ or facility. We advise every dentist to read the article just refered to.

Now we wish to direct the attention of the profession to the fact that the first of August is drawing near, and the committee on the Barnum fund are becoming quite anxious in view of the receipts up to this time. Now let all fully appreciate the importance of this matter and send on their contributions to either member of the committee at once. And let this acknowledgment be of such an amount, and with such a spirit of appreciation as shall bear some sort of proportion to the boon conferred.

